# Effect of Deep Drying and Torrefaction Temperature on Proximate, Ultimate Composition, and Heating Value of 2-mm Lodgepole Pine (*Pinus contorta*) Grind

**DOI:** 10.3390/bioengineering3020016

**Published:** 2016-06-22

**Authors:** Jaya Shankar Tumuluru

**Affiliations:** Idaho National Laboratory, 750 University Blvd., Energy Systems Laboratory, PO: Box: 1625, Idaho Falls, ID 83415, USA; jayashankar.tumuluru@inl.gov; Tel.: +1-208-526-0529

**Keywords:** lodgepole pine, torrefaction, thermogravimetric analysis, proximate and ultimate composition

## Abstract

Deep drying and torrefaction compose a thermal pretreatment method where biomass is heated in the temperature range of 150–300 °C in an inert or reduced environment. The process parameters, like torrefaction temperature and residence time, have a significant impact on the proximate, ultimate, and energy properties. In this study, torrefaction experiments were conducted on 2-mm ground lodgepole pine (*Pinus contorta*) using a thermogravimetric analyzer. Both deep drying and torrefaction temperature (160–270 °C) and time (15–120 min) were selected. Torrefied samples were analyzed for the proximate, ultimate, and higher heating value. The results indicate that moisture content decreases with increases in torrefaction temperature and time, where at 270 °C and 120 min, the moisture content is found to be 1.15% (w.b.). Volatile content in the lodgepole pine decreased from about 80% to about 45%, and ash content increased from 0.77% to about 1.91% at 270 °C and 120 min. The hydrogen, oxygen, and sulfur content decreased to 3%, 28.24%, and 0.01%, whereas the carbon content and higher heating value increased to 68.86% and 23.67 MJ/kg at 270 °C and 120 min. Elemental ratio of hydrogen to carbon and oxygen to carbon (H/C and O/C) calculated at 270 °C and a 120-min residence time were about 0.56 and 0.47. Based on this study, it can be concluded that higher torrefaction temperatures ≥230 °C and residence time ≥15 min influence the proximate, ultimate, and energy properties of ground lodgepole pine.

## 1. Introduction

In the recent United Nations Paris Framework Convention on Climate Change (PFCCC), there is a call to mitigate the global annual emissions of greenhouse gases by 2020 in order to reduce the global average temperature increase to less than 2 °C [1]. Renewable energies represent a diversity of sources that can help to maintain the equilibrium of different ecosystems. Among these, biomass is considered carbon neutral due to the carbon dioxide released during its conversion that is already part of the carbon cycle [2]. According to the Kyoto Protocol [3], increasing the use of biomass helps to reduce carbon dioxide emissions and to reduce its negative impact on the environment. According to the U.S. Department of Energy (DOE) [4], about a billion tons of biomass is available in the United States for energy applications. This enhances the ability of the United States to include biomass as a sustainable and significant part of domestic energy production.

### 1.1. Biomass Limitations for Solid and Liquid Fuel Applications

Energy from biomass can be produced using thermochemical (direct combustion, gasification, and pyrolysis), biological (anaerobic digestion and fermentation), and chemical (esterification) technologies. Out of all of these technologies, combustion of biomass can provide a direct near-term energy solution. The inherent physical (particle size and density) and chemical characteristics (proximate, ultimate, and energy properties) of raw biomass restrict its use in higher percentages for direct-combustion applications. Furthermore, grinding raw biomass with high moisture content is very challenging due to its fibrous nature. The study of Tumuluru *et al*. [5] indicated that high moisture in biomass increases the grinding energy and negatively impacts the particle size distribution. Additionally, higher moisture in the biomass will result in plugging the grinder screens and the reactor and bridging of the particles in the conveyors. In terms of chemical composition, the raw biomass has a higher oxygen and hydrogen content and a lower carbon and calorific value as compared to fossil fuels [6]. All of these limitations do not make biomass a great candidate for production of solid and liquid biofuels. In their studies, Tumuluru *et al*. [7] researched the impact of feedstock supply system unit operations and the effects on feedstock quality and cost. The same authors suggested that new harvesting methods and mechanical, chemical, thermal pretreatment technologies will help to improve the biomass physical and chemical properties. These improvements will make biomass meet the specifications in terms of density, particle size, ash composition, and carbohydrate content for both biochemical and thermochemical conversion applications. Various thermal methods, such as dry and wet torrefaction (hydrothermal carbonization) and steam explosion, as well as chemical pretreatment techniques, such as ionic, acid, alkali, and ammonia fiber expansion, were investigated to understand how they improve the biomass specifications for biofuels production [8,9,10,11,12,13]. Recent studies by Sarkar *et al*. [14] and Yang *et al*. [15] on using torrefied and torrefied-densified switchgrass for both gasification and pyrolysis applications have indicated that higher quality syngas and bio-oil are produced when compared to the ones produced using raw switchgrass. In their studies, Hoover *et al*. [16], Ray *et al*. [17], and Aston *et al*. [18] indicate that chemical pretreatments (*i.e.*, ammonia fiber expansion (AFEX), acid, and alkali) and further densification help to increase the liquid fuels production through biochemical conversion.

### 1.2. Dry Torrefaction

**Figure 1:**
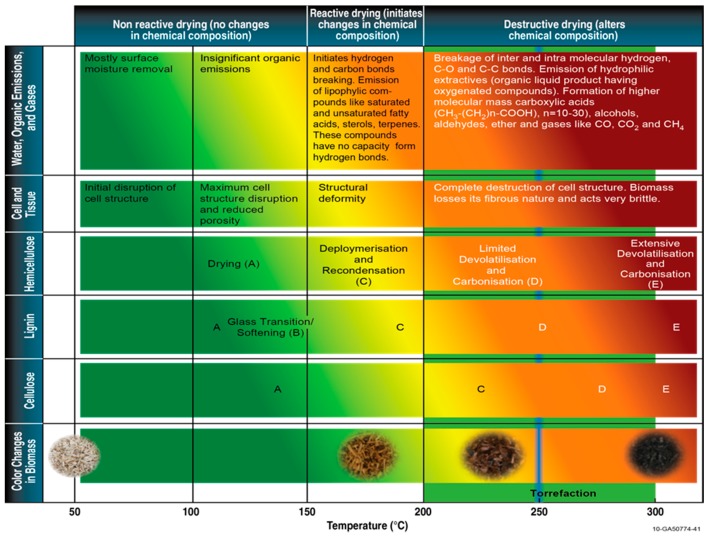
Non-reactive, reactive, and destructive drying temperature impact on the biomass components (adapted from [13]).

Dry torrefaction is a thermal pretreatment technique that is used to improve the physical and chemical properties of biomass. Torrefaction is defined as slowly heating the biomass in an inert atmosphere to a maximum temperature of 300 °C [19,20], which will result in a solid uniform product with lower moisture and higher energy content when compared to raw biomass. Various biomass reactions occur during torrefaction, such as devolatilization and carbonization of hemicellulose, depolymerization and devolatilization/softening of lignin and depolymerization, and devolatilization of cellulose. These reactions result in changes in biomass moisture, chemical composition, and energy content. The feedstock and process variables that influence the torrefaction process are (1) feedstock type, (2) particle size, (3) temperature, (4) residence time, and (5) heating rate [13]. Figure 1 indicates the change in the biomass components at different temperature regimes [13].

Tumuluru *et al*. [13] divide thermal pretreatment regimes into three zones: non-reactive 50–150 °C, reactive 150–200 °C, and destructive drying 200–300 °C. According to Tumuluru and Hess [21] and Tumuluru *et al*. [13], the reactive drying temperature regime is also called deep drying, whereas the destructive drying temperature is called the torrefaction regime. The initial heating of biomass up to 150 °C (non-reactive drying zone) removes the unbound water. Increasing the temperature from 150–200 °C removes most of the bound water by a thermo-condensation process [22]. Furthermore, increasing the temperature to >200 °C will result in decomposition, devolatilization, and carbonization reactions. Most of the biomass hemicellulose undergoes decomposition reactions, which result in significant changes in color, chemical composition, and physical characteristics. At temperatures >280 °C, the process is completely exothermic, which results in significant increases in the production of CO_2_, phenols, acetic acid, and other higher hydrocarbons. During torrefaction, hemicellulose degradation causes the destruction of OH functional groups and will leave the material hydrophobic. The degree of hydrophobicity depends on the torrefaction temperature. Hydrophobicity will help biomass to absorb less moisture during its storage in different environments, therefore making it more stable against fungal and microbial attacks.

### 1.3. Deep Drying and Torrefaction Parameters

The biomass parameters commonly used in deep drying and torrefaction are reaction temperatures of 160–300 °C, heating rates of <50 °C/min, and residence times <30 min [13]. The absence or limiting of oxygen presence to <7% in the reactor by avoiding oxidation and auto-ignition during its heating is of great importance. Many researchers have worked on the torrefaction of agricultural and woody biomass using a thermogravimetric analyzer (TGA) [6,23,24]. These researchers have successfully used TGA, on both woody and herbaceous biomass, to understand the effect of temperature and residence time on torrefaction kinetics and the chemical, proximate, and energy properties. Tumuluru [6] studied torrefaction of corn stover and switchgrass using TGA, concluding that the torrefaction temperature has the most significant effect compared to residence time on the chemical and energy properties.

### 1.4. Objectives

In the past several decades, lodgepole pine (*Pinus contorta*) has gained much importance due to its high quality timber and yields [25]. This is the third largest timber type in the Western United States after ponderosa pine and Douglas fir. Lodgepole pine is available in larger quantities, which leads to a growing interest in its suitability as a feedstock for bioenergy applications [25,26,27]. In the present study, lodgepole pine was selected to understand the changes torrefaction can bring in terms of the proximate, ultimate, and energy properties. In the present research, a smaller particle size of 2 mm was selected to conduct the torrefaction studies. Studies conducted by Bridgewater *et al*. [28] indicate that for efficient thermo-chemical conversion, such as pyrolysis, particle sizes of approximately 2-mm are necessary. In general, bigger particle sizes result in secondary reactions, leading to the formation of more tars, whereas smaller particle sizes due to less time in the reaction zone result in minimizing the secondary reactions and, in turn, result in less tar formation.

The overall objective of this work is to conduct thermal pretreatment studies (deep drying and torrefaction) over a wide range of temperatures and residence times using the TGA. The specific objectives of this study are (1) to understand the effect of deep drying and torrefaction temperature in the range of 160–270 °C and a residence time of 15–120 min on the proximate composition (*i.e.*, moisture content, volatile content, fixed carbon content, and ash content), the ultimate composition (*i.e.*, carbon, hydrogen, sulfur, nitrogen, oxygen, and H/C and O/C ratios), and the higher heating value of a 2-mm lodgepole pine grind; (2) to understand the significance of the torrefaction temperature and the residence time with respect to the ultimate composition, proximate composition, and higher heating values; and (3) to develop linear regression models for the torrefaction parameters with respect to the proximate composition, ultimate composition, and higher heating values.

## 2. Materials and Methods

Clean lodgepole pine (*Pinus contorta*) wood chips were used in the present study. The wood chips were dried to <5% (w.b.) moisture content in a laboratory oven and were further ground in a Willey mill fitted with a 2-mm screen. The ground samples were double bagged, stored in an air-tight container, and used to conduct the torrefaction tests.

### 2.1. Torrefaction Studies Using the Thermogravimetric Analyzer

**Figure 2:**
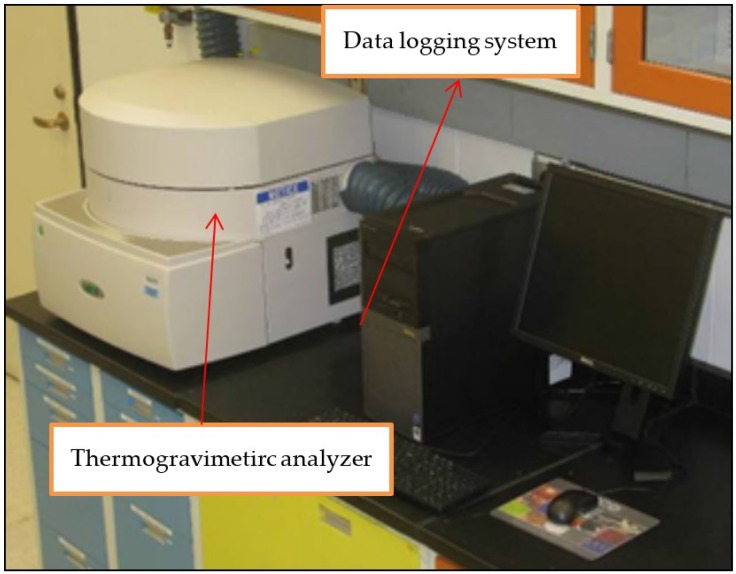
LECO Model 701 thermogravimetric analyzer [6].

Deep drying and torrefaction studies on ground lodgepole pine was carried out using TGA (Figure 2). The LECO (Model No: TG 701) TGA was used to conduct the torrefaction tests. A N_2_ atmosphere was used in this study. A method file was developed to carry out the torrefaction studies using TGA [24]. TGA also measures mass loss as total moisture, ash, volatile content, or loss-on-ignition. The LECO C/H/N analyzer was used for estimating the carbon, hydrogen, and nitrogen of the raw and torrefied samples. The American Society for Testing and Materials (ASTM standard) methods were used for estimating the chemical composition (Table 1). The higher heating values of raw and torrefied samples were measured using a bomb calorimeter. The reported chemical composition and energy property are an average of three measurements. Experiments were conducted in a temperature range of 160–270 °C and residence times of 15–120 min. Table 2 indicates the experimental design followed for conducting deep drying and torrefaction studies.

### 2.2. Data Analysis: Analysis of Variance and Multiple Regression Analysis

The experimental data on the proximate composition, ultimate composition, and higher heating values obtained at different torrefaction temperatures (160, 180, 230 and 270 °C) and residence times (15, 30, 60 and 120 min) for a 2-mm lodgepole pine grind were used to draw the linear plots, develop multiple regression models, (Equation 1), and understand the significance of the torrefaction process variables with respect to chemical properties that were studied. Dell Statistica (Version 10) statistical software was used to develop the multiple regression models and analysis of variance (ANOVA) of the experimental data. The coefficient of determination was used to determine the model fit.

(1) $$f\left(y\right)=b_{0}+b_{1}x_{1}+b_{2}x_{2}$$

where:

$$b_{0},b_{1}{\text{ and b}}_{2}=\text{equation constants}$$

$$x_{1}\text{ and }{\text{x}}_{2}\;=\text{ torrefaction temperature and torrefaction residence time}$$

## 3. Results

**Figure 3:**
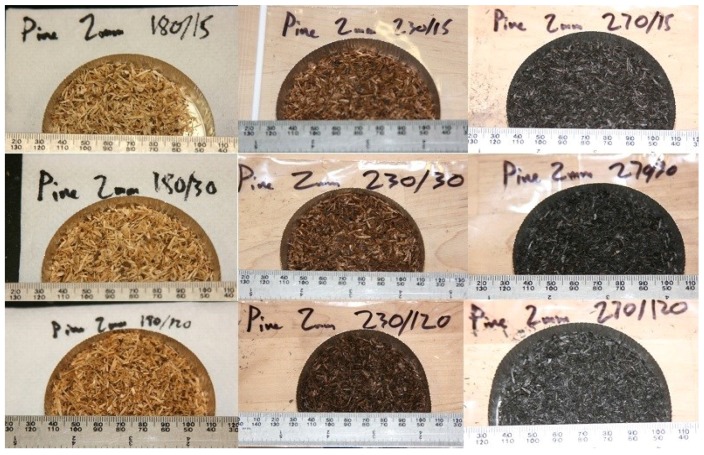
Color changes in torrefied lodgepole pine grind at different torrefaction temperatures and residence times.

Figure 3 indicates the ground lodgepole pine samples that were torrefied at different temperatures and residence times. It is clear from the figure that when increasing the torrefaction temperature, the color of the biomass changes from brown to black. Tumuluru *et al*. [13] report that the biomass turns brown to black at 150–300 °C. This can be mainly attributed to the chemical compositional changes that occur in the biomass components, such as hemicellulose, lignin, and cellulose. The major reactions that change the biomass color are devolatilization and carbonization of hemicellulose, depolymerization and devolatilization/softening of lignin, and depolymerization and devolatilization of cellulose. Food industries use color as an indicator to understand the quality changes in products. For example, in a coffee-bean roasting process, the change in color is used as an indicator to define the degree of roasting. The chemical compositional changes are due to the breakage of hydrogen and carbon bonds. The disruption of most inter- and intra-molecular hydrogen bonds, C–C and C–O bonds, results in the formation of hydrophilic extractives, carboxylic acids, alcohols, aldehydes, ether, and gases (e.g., CO, CO_2_, and CH_4_). All of these chemical compositional changes within the biomass will significantly change its color. Branca *et al*. [36] indicate that the color of the wood chips changes when the biomass is torrefied at different temperatures and residence times. Nhuchhen *et al*. [37] observed a similar color change, where higher torrefaction temperatures resulted in a dark black product.

### 3.1. Proximate, Ultimate Composition and Higher Heating Values

Proximate-composition measurement includes moisture content, volatiles’ content, ash content, and fixed carbon, whereas fixed carbon is calculated based on the difference. The ultimate composition includes carbon, hydrogen, nitrogen, sulfur, and oxygen and the oxygen content is calculated by the difference method. The proximate, ultimate, and higher heating values of the 2-mm raw lodgepole pine grind are shown in Table 3.

### 3.2. Moisture Content

**Figure 4:**
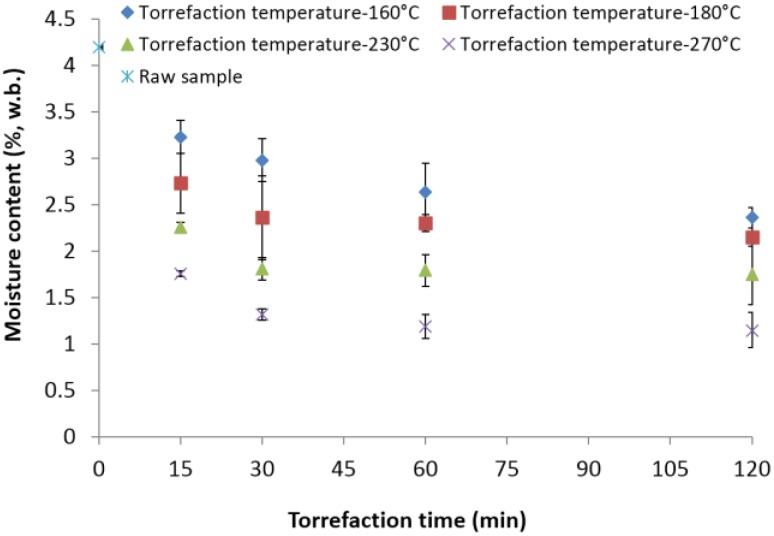
Moisture content in the lodgepole pine grind with respect to the torrefaction temperature and time.

The initial moisture content of the 2-mm lodgepole pine grind was about 4.2% (w.b.). After torrefaction, the torrefied samples’ moisture content had significantly decreased with the increase in torrefaction temperature and time. The lowest moisture content observed was about 1.15% (w.b.) at 270 °C and a 120-min residence time (Figure 4). The percent decrease observed in moisture content was about 23.09% from the initial value of about 4.2% (w.b.) to 3.23% (w.b.) at 160 °C for 15 min. Increasing the torrefaction temperature and time to 270 °C and 120 min, the moisture content of the samples reduced by about 72.61% from the initial value of 4.2%. The decrease in moisture content from 160, 180, 230 and 270 °C and a 30-min residence time was about 29.04%, 43.80%, 56.90% and 68.57%. A similar decrease in moisture content was observed at other residence times, as well.

### 3.3. Volatile Content

**Figure 5:**
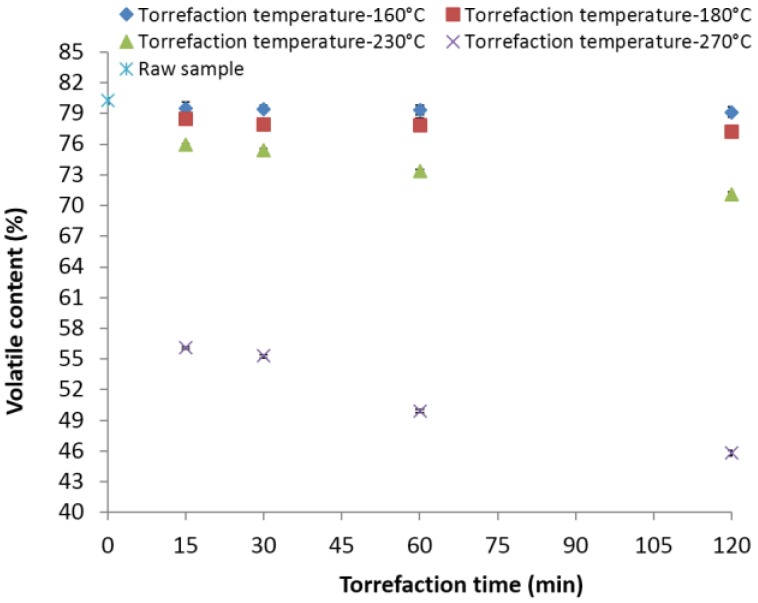
Volatile content in the lodgepole pine grind with respect to the torrefaction temperature and time.

At lower torrefaction temperatures (160 and 180 °C) and different residence times, the decrease in volatile content in the torrefied biomass was relatively minimal (the maximum decrease was about 1.3% and 3.72% at 120 min with respect to the original value) (Figure 5). Increasing the torrefaction temperature to 230 and 270 °C and its residence time to 120 min reduced the volatile content to about 75.41% and 55.28% compared to its original value. The effect of torrefaction temperature showed a more significant effect when compared to the residence time. Carter *et al*. [38] reported similar volatile content (76.37% and 56.53%) in the lodgepole pine samples when torrefied at 225 and 275 °C for a 30-min residence time.

### 3.4. Ash Content

**Figure 6:**
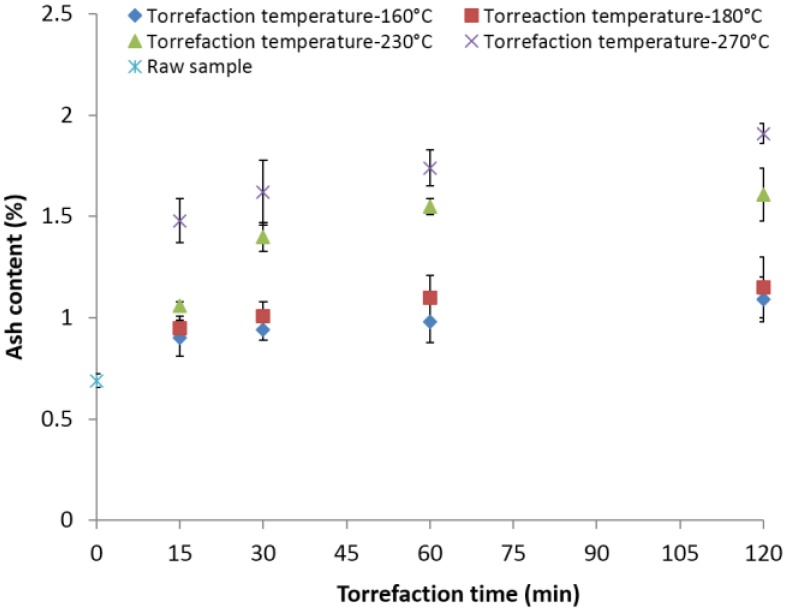
Ash content in the lodgepole pine grind with respect to the torrefaction temperature and time.

The ash content in the torrefied material increased with the increase of torrefaction temperature and time (Figure 6). The changes in the ash content are mainly due to breakdown of carbon-hydrogen bonds, resulting volatile loss and further concentrating the ash content in the biomass. The highest ash content of about 1.91% was observed for samples torrefied at 270 °C and 120 min. The increase was about 176% with respect to the original value. The increase in relative ash content at different torrefaction temperatures (160, 180, 230 and 270 °C) and a 30-min residence time was 36.9%, 46.37%, 102.01% and 134.78% compared to its initial value of 0.69%.

### 3.5. Fixed Carbon

**Figure 7:**
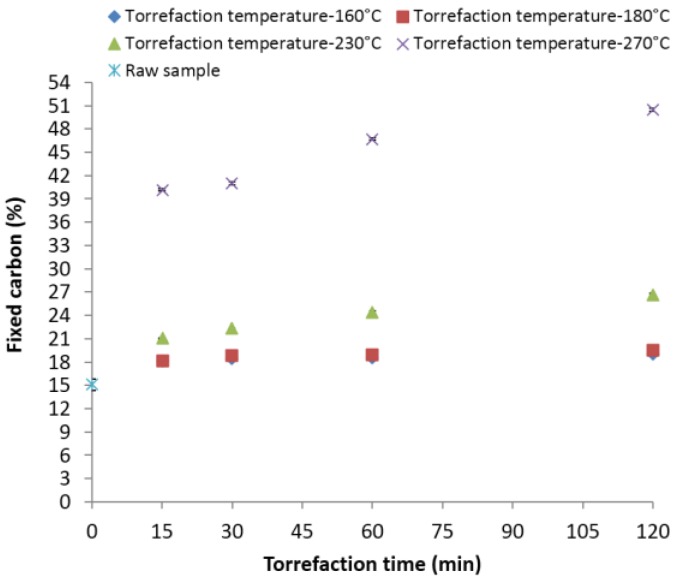
Fixed carbon content in the lodgepole pine grind with respect to the torrefaction temperature and time.

Figure 7 indicates the changes in the fixed carbon content in the ground lodgepole pine torrefied at different temperatures and residence times. The initial fixed carbon of the raw biomass was 15.91%. Increasing the temperature to 160, 180, 230 and 270 °C and a 30-min residence time increased the fixed carbon content to about 18.43%, 18.79%, 22.34% and 41.01% (an increase of 15.83%, 18.01%, 40.41%, and 157.76% from its original value). The maximum fixed carbon observed at 270 °C and 120 min was about 50.48% (Figure 7). Fixed carbon content data matched closely with the data presented by Carter *et al*. [38], where at 225 and 275 °C for 30 min, the observed values were 22.75% and 42.49% for lodgepole pine. The results indicate that the increase is marginal at 160 and 180 °C at all residence times, but further increasing the temperature to 230 and 270 °C significantly increased the fixed carbon content.

### 3.6. Ultimate Composition

Ultimate composition measurements include elemental carbon, hydrogen, sulfur, nitrogen, and oxygen by different methods. The ultimate composition of the 2-mm ground lodgepole pine raw material is 6.2% hydrogen, 52.3% carbon, 0.28% nitrogen, 0.02% sulfur, and 41.23% oxygen.

### 3.7. Carbon (%)

**Figure 8:**
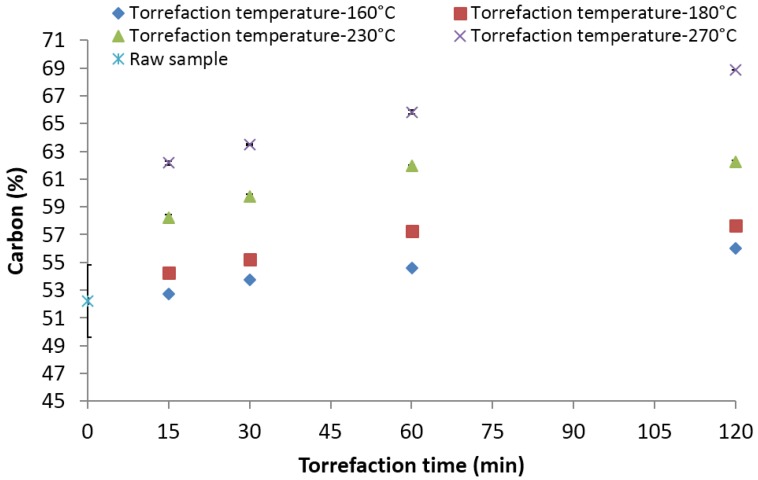
Carbon content in the lodgepole pine grind with respect to the torrefaction time and temperature.

The initial elemental carbon in the 2-mm ground lodgepole pine sample was about 52.23%. Heating the biomass at 160 and 180 °C for a 30-min residence time increased the elemental carbon content to about 56%–57% (Figure 8). Further, increasing the temperature and time to 230 and 270 °C for 120 min increased the carbon content to about 62% and 68%. It is clear from the data that the increase in elemental carbon is significantly higher at temperatures ≥230 °C. It is also clear from the data that the increase in elemental carbon with the increase in residence time (from 15 to 120 min) is about 3%, whereas increasing the torrefaction temperature from 230 to 270 °C resulted in about an 8% rise for the residence times studied. Carter *et al*. [38] reports similar values of carbon content (64.17%) for pine when torrefied at 275 °C and a 30-min residence time.

### 3.8. Hydrogen (%)

**Figure 9:**
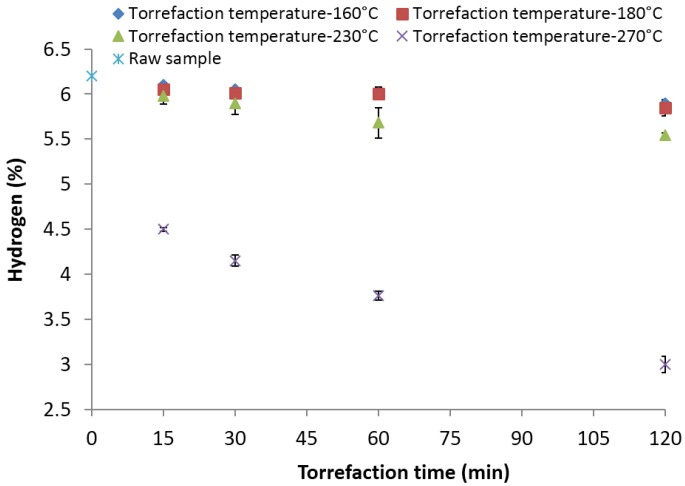
Hydrogen content in the lodgepole pine grind with respect to the torrefaction time and temperature.

The hydrogen content of the raw biomass samples observed was about 6.2%. The decrease in hydrogen content at 160 and 180 °C for different residence times was marginal, where a maximum decrease of about 5.85% was observed at 180 °C and 120 min (Figure 9). Increasing the torrefaction temperature and residence time to 230 and 120 min reduced the hydrogen content to about 5.54 and 3%, respectively. The results indicate that the decrease is more significant at higher temperatures of >230 °C. Furthermore, the data from the present study indicate that higher torrefaction temperatures and residence times played a major role in reducing the hydrogen content. At 270 °C and a 15-min residence time, the hydrogen content observed was 4.5%, whereas at a 120-min residence time, the final hydrogen content observed is about 3% at 270 °C. Carter *et al*. [38] observed similar trends where hydrogen was about 4.81% at 275 °C and 30 min; further increasing the time to 45 min reduced the hydrogen content to 3.47%.

### 3.9. Oxygen (%)

**Figure 10:**
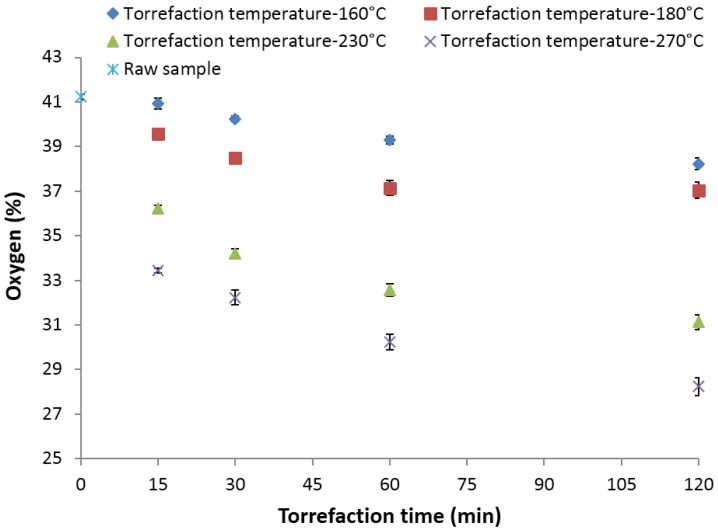
Oxygen content in the lodgepole pine grind with respect to the torrefaction time and temperature.

The oxygen content of the raw and torrefied samples was calculated based on the difference method. The initial oxygen content of the raw samples observed was 41.23%. At 160 and 180 °C, the oxygen content observed in the samples was in the range of 40.93%–37.04%, whereas at 230 °C, the oxygen content reduced to about 31.14% (a decrease of about 24.47% from the initial value). Further increasing the torrefaction temperature to 270 °C and the residence time to 120 min decreased the oxygen content in the sample to about 28.24% (a decrease of about 32% from the initial value). The data presented by Carter *et al*. [38] for oxygen content in the pine samples when torrefied at 275 °C for a 30-min residence time was 29.27%, which matched closely with the values reported in this research (32.24%). At 160, 180, 230, and 270 °C and a 30-min residence time, the observed oxygen values were 40.24%, 38.49%, 34.21% and 32.24% (Figure 10). The results indicate that torrefaction temperature had a higher impact on the oxygen content compared to the residence time.

### 3.10. Nitrogen and Sulfur (%)

The initial nitrogen and sulfur content observed for the raw biomass samples was 0.47% and 0.02%. At lower temperatures of 160 and 180 °C and different residence times, the changes in nitrogen and sulfur content were marginal; however, increasing the torrefaction temperature to 230 and 270 °C at 30 min decreased the nitrogen and sulfur content to 0.25% and 0.015%. Furthermore, increasing the residence time to 120 min decreased the nitrogen and sulfur content to final values of 0.17% and 0.01%.

### 3.11. H/C and O/C Ratios

**Figure 11:**
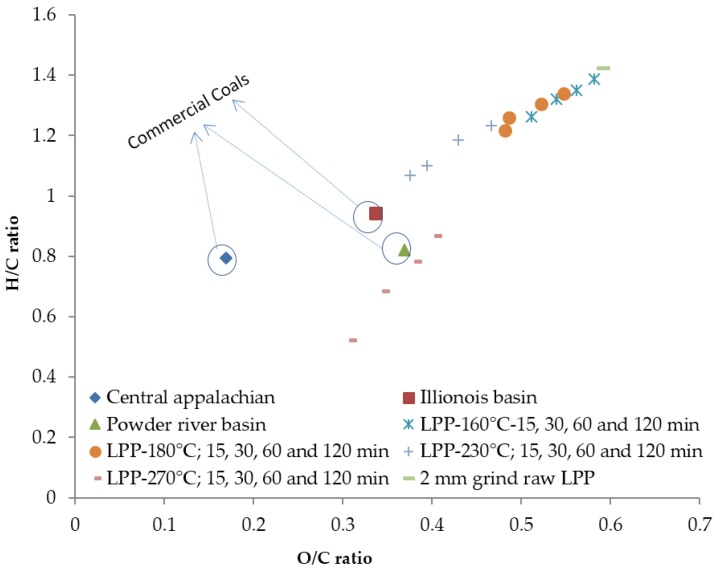
van Krevelen diagram for the raw, torrefied lodgepole pine grind, and the commercial coals.

The initial hydrogen to carbon (H/C) and oxygen to carbon (O/C) ratios of the lodgepole pine sample were 1.42 and 0.59. The H/C ratio decreased when the torrefaction temperature and residence time was increased. The lowest H/C and O/C ratios observed were 0.56 and 0.47 at a torrefaction temperature of 270 °C and a 120-min residence time (Figure 11). At 160 and 180 °C and a 30-min residence time, the H/C ratio was about 1.31 and 1.28 and the O/C ratio was about 0.99 and 0.69. Increasing the torrefaction temperature to 230 and 270 °C and the residence time to 30 min decreased the H/C ratio to 1.19 and 0.88 and the O/C ratio to near 0.70 and 0.41. Further increasing the residence time to 120 min at the same temperature decreased the H/C and O/C ratios. The change in the O/C ratio with respect to the torrefaction temperature is more significant than the torrefaction residence time.

### 3.12. van Krevelen Diagram

The van Krevelen diagram was drawn for the O/C and H/C ratios, for the raw and torrefied ground lodgepole pine, and is compared to different grades of commercially available coals (Central Appalachian, Illinois Basin, Powder River Basin) (Figure 11). According to this plot, torrefaction of ground lodgepole pine shifts the elemental ratios of H/C and O/C (0.78 and 0.36) closer to the commercially available coals, such as Illinois Basin and Powder River Basin coals. Figure 11 indicates that the high-quality coals have a lower ratio of H/C to O/C (mainly due to the lower oxygen content and higher carbon content) compared to the ground lodgepole pine. Torrefaction of lodgepole pine at 160, 180 and 230 °C at different residence times advanced the H/C and O/C ratios closer to the commercial coals. At a 270 °C torrefaction temperature and a 15–120-min residence time, the ground lodgepole pine moved close to Power River Basin and Illinois Basin coal. There was a major shift in the H/C and O/C values from 230 to 270 °C (Figure 11). The shift is mostly due to the significant increase in carbon content and the steep decrease in oxygen and hydrogen content. The breakage of inter- and intra-molecular hydrogen and carbon-oxygen and carbon-carbon bonds result in the emission of extractives and oxygenated compounds, which might have resulted in the major shift in the H/C and O/C values. Additionally, Dutta [39] reports similar elemental ratios of the H/C and O/C ratio values when pine was torrefied at a higher temperature of 270 °C and a 30-min residence time.

### 3.13. Higher Heating Value (MJ/kg)

**Figure 12:**
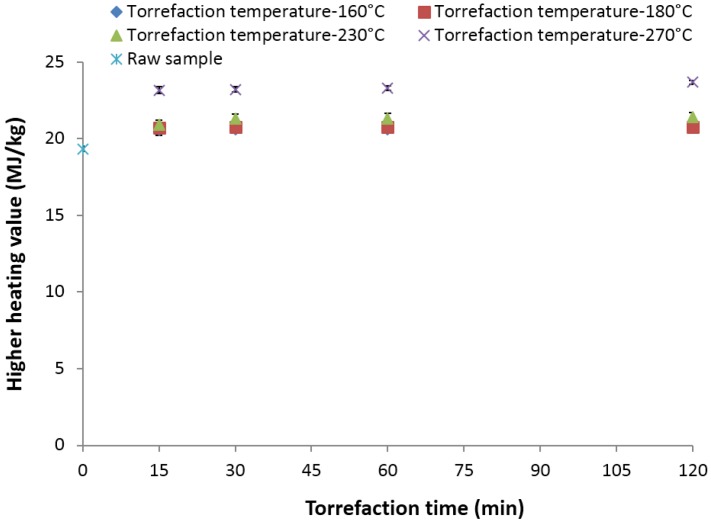
Higher heating value in the lodgepole pine grind with respect to the torrefaction time and temperature.

The initial higher heating value observed for the 2-mm ground lodgepole pine was 19.45 MJ/kg. Increasing the torrefaction temperature to 270 °C and the time to 30 min significantly increased the heating value to about 23.67 MJ/kg (Figure 12). At lower temperatures of 160 and 180 °C and a 120-min residence time, the heating value increase was found to be marginal (there is about a 1 MJ/kg increment). Increasing the torrefaction temperature from 180 to 230 °C for residence times of about 30 min, the increase was about 2 MJ/kg (21.34 MJ/kg), whereas at 270 °C, the increase was about 4 MJ/kg (23.21 MJ/kg) compared to the higher heating value of raw lodgepole pine grind. The maximum higher heating value observed at 270 °C and a 120-min residence time was about 21.57 MJ/kg. Peng *et al*. [40] report that torrefying lodgepole pine at 250 and 300 °C at 30 min results in higher heating values in the range of 20.58–23.02 MJ/kg.

## 4. Analysis of Variance and Multiple Regression Models

The experimental data were further analyzed to understand the significance of the process variables, with respect to the proximate and ultimate composition and higher heating values. Multiple regression models were also developed for proximate, ultimate composition, and energy data. Table 4 shows the multiple regression equations fitted for the experimental data, and Table 5 indicates the significance of the process variables based on the ANOVA. The regression equations developed were found to be statistical significant at *p* < 0.001. The regression equations indicated that in the case of proximate composition, the torrefaction temperature and residence time were positively correlated for fixed carbon and ash content, whereas for moisture and volatiles, there was a negative correlation. Furthermore, the regression equations indicated that the coefficient of torrefaction temperature was higher when compared to the torrefaction residence time. In the case of the ultimate composition, hydrogen, nitrogen, oxygen, sulfur, the H/C ratio, and the O/C ratio were negatively correlated, whereas in the case of carbon content and higher heating values, they are positively correlated with the torrefaction temperature and residence time.

ANOVA analysis indicates that the moisture content, ash content, and fixed carbon were influenced by both the torrefaction temperature and residence time at *p* < 0.001. In the case of volatile content, the torrefaction temperature, at *p* < 0.001, and not by residence time, had a significant effect. In the case of the ultimate composition, hydrogen content was influenced by torrefaction temperature at *p* < 0.001, whereas residence time was found to be insignificant. Carbon, oxygen, and sulfur content were influenced by both torrefaction temperature and residence time at *p* < 0.001, whereas nitrogen content was influenced by torrefaction time at *p* < 0.01. The H/C and O/C ratios are influenced by the torrefaction temperature at *p* < 0.001, whereas the residence time influenced the H/C ratio at *p* < 0.05 and not the O/C ratio. In the case of a higher heating value, the torrefaction temperature was influenced at *p* < 0.001, and the residence time was not significant.

Bates and Ghoniem [41] indicate that torrefaction temperatures of 250 and 300 °C and residence times of 15–60 min result in 16%–30% solid mass loss; increasing the residence time further results in an additional 42%–48% mass loss. They have concluded that mass loss is faster in the first stage of torrefaction, which is primarily attributable to the decomposition of hemicellulose (with an increasing contribution from cellulose decomposition at higher temperatures). The non-condensable products, such as CO_2_ and CO, reach the peak value at residence times of 10 min and then start to decline. The same authors also indicate that the amount of methanol and lactic acid, which is produced during the decomposition of acetoxy- and methoxy-groups, increased up to 10 min, and then it remained unchanged. The observations in this study indicate that the coefficient of torrefaction temperature is higher when compared to the torrefaction residence time. Additionally, the analysis of variance supports this observation, where torrefaction temperature has more significant impacts on the proximate and ultimate composition and its higher heating value when compared to torrefaction residence time. Nhuchhen [37] work on torrefaction of biomass indicate that the net effect of the residence time is not as prominent as the temperature; the same authors also concluded that in a rotary torrefaction system, the torrefaction temperature is more prominently impactful when compared to angular speed and the inclination of the dryer.

## 5. Discussion

The moisture content of lodgepole pine biomass reduces both at deep drying and torrefaction temperatures. If the target is to reduce the moisture content of the biomass, deep drying at 160 and 180 °C for <30 min will help to reduce most of the moisture in the lodgepole pine. At deep drying temperature, the loss of moisture content can be due to dehydration reactions with less change to the chemical composition of the biomass. At higher torrefaction temperatures between ≥180 and ≤350 °C, the loss of moisture and volatiles can be due to the devolatilization and carbonization of hemicellulose and cellulose [24]. Bergman and Kiel [19] and Prins [42] report that drying and depolymerization occur between 225 and 325 °C for hemicelluloses. According to Bridgeman *et al*. [24], the loss of moisture is due to the evaporation and dehydration reactions between the organic molecules, which result in the release of organic and inorganic products from the biomass. The organics that are typically released during torrefaction include sugars, poly-sugars, acids, alcohols, furans, and ketones [13]. The present study indicated that the change in the chemical composition at deep drying temperature (160 and 180 °C) is minimal compared to torrefaction temperatures of 230 and 270 °C.

According to Tumuluru *et al*. [13], at deep drying temperatures of 160 and 180 °C, the changes in the biomass are due to dehydration reactions, whereas at torrefaction temperature of >230 °C, the major reactions are devolatilization and carbonization, causing significant changes in the chemical composition when compared to the raw material. Bates and Ghoniem [41], in their study, indicate that at a lower temperature of 200 °C, the composition is similar to non-torrefied willow, whereas at an increased temperature, it will increase the mass fraction of carbon, while those of hydrogen and oxygen decrease. The authors’ two-step model indicates that in the first stage, oxygenated species, such as water, acetic acid, and carbon dioxide, are released, and at the second stage, lactic acid, methanol, and acetic acid were released. Medic *et al*. [43], in their studies on the effects of torrefaction process parameters on biomass feedstock upgrading, conclude that corn stover undergoes changes in chemical composition and energy content during the torrefaction process. These authors also indicate that at higher torrefaction temperatures, the biomass can be characterized in several ways, such as a mass loss of up to 45%, a decrease in the O/C ratio from 1.11 to 0.6, and an increase in the energy density of about 19%.

Tumuluru *et al*. [13], in their review on the torrefaction of biomass, indicate that at >250 °C, the biomass undergoes extensive devolatilization and carbonization. These reactions result in the loss of volatiles and an increase in the carbon content. Furthermore, it is observed that the change in the ash content is related to the loss of other biomass components, such as moisture and volatiles. According to Park *et al*. [44], during torrefaction, volatile loss is due to thermal breakdown of carbohydrate fractions, which results in the accumulation of the residual ash after torrefaction. The same authors further indicate that these changes increase the fixed carbon content of the biomass. Ultimate composition data indicate that oxygen is reduced during torrefaction and the reduction is increased at higher torrefaction temperatures. In the present study, changes in hydrogen and oxygen were marginal at a lower temperature of 180 °C, when compared to torrefaction temperatures of 230 and 270 °C. This observation is corroborated with the findings of other researchers [13,45,46,47]. The decrease of oxygen content is mainly due to the dehydration reaction, which produces water vapor and releases CO and CO_2_. During torrefaction, the loss of volatiles, gases, and water also results in a decrease in hydrogen content. The decrease in oxygen and hydrogen content and the increase of carbon content (from approximately 51%–66%), at a 270 °C torrefaction temperature and a 30-min residence time, results in lowering the atomic O/C ratio (from 0.63 to 0.31). According to Tumuluru *et al*. [46], torrefaction temperatures of >300 °C may not be needed, because there is a significant loss of higher energy content volatiles. Furthermore, it may increase the relative ash content of the biomass.

According to the van Krevelen diagram, the substantial change in the atomic ratio of carbon, hydrogen, and oxygen makes torrefied material act more like coal, making the torrefied material more suitable for co-firing applications. Lodgepole pine torrefied at 270 °C and at different residence times reduced the H/C and O/C ratios and moved them closer to high-quality coal, such as Powder River Basin and Illinois Basin coals. The lower O/C ratio observed at higher torrefaction temperatures can be due to the release of oxygen-rich compounds (e.g., CO, CO_2_, and H_2_O), whereas lower H/C ratios can be due to the formation of CH_4_ and C_2_H_6_ during torrefaction. The decrease of the H/C and O/C ratios with an increase in the torrefaction temperature and residence time results in fuels that produce less smoke, less water-vapor formation, and lower energy loss during the combustion and gasification processes [6].

The heating value of biomass is an important quality attribute for energy generation applications [2]. The increase in torrefaction temperature and residence time increases the calorific value (higher heating value), and higher temperatures and residence times result in the loss of more volatiles and increased energy density. The increase in the heating value is due to the decrease in moisture content and an increase of carbon content in the samples. Zanzi *et al*. [22] and Nimlos *et al*. [48], in their report, confirm that increasing the torrefaction temperature increases the higher heating value. Tumuluru *et al*. [12] indicate that at torrefaction temperatures of >300 °C, there is a significant loss of higher energy content volatiles within the biomass.

The feedstock variable that impacts the proximate, ultimate, and energy properties of the torrefied material is the particle size used for torrefaction. Smaller particle sizes used in the present study might have been more reactive to torrefaction temperature due to the higher surface area. According to Nhuchhen [49] and Wang *et al*. [50], particle size impacts the devolatilization reactions. These authors further indicate that a smaller particle size results in a greater intra-particle effect and heat transfer. Another important torrefaction parameter that can impact the resolution of the experimental data is the ramp time. This can impact the torrefaction reaction kinetics of the various components, such as lignin, hemicellulose, and cellulose. Current studies indicate the torrefaction of ground lodgepole pine at 270 °C and a 15–30-min residence time improves the chemical composition and the higher heating value, making lodgepole pine more suitable for bio-power generation.

## 6. Conclusions

The research presented was carried out to understand the effect of torrefaction temperature and residence time on the proximate composition, ultimate composition, and higher heating values of a 2-mm lodgepole pine grind. Based on this research the following conclusions are drawn:

- The changes in proximate and ultimate composition were marginal at deep drying temperatures of 160 and 180 °C, whereas at torrefaction temperatures of 230 and 270 °C, the changes were significant.
- Increasing the torrefaction temperature to 270 °C and the residence time to 30 min significantly decreases the moisture content, hydrogen content, and oxygen content and increases the carbon and heating value.
- At 270 °C and a 120-min residence time, carbon content increases to 69.86%, while oxygen and hydrogen content decrease to 28.24% and 3%, whereas the volatile content decreases to 45.81%.
- The H/C and O/C ratios of the raw samples are about 1.42 and 0.59, whereas at 270 °C and 120 min, the H/C and O/C ratio decreases to 0.56 and 0.47.
- The changes in these chemical compositions are attributed to the devolatilization of hemicellulose, which will typically happen at torrefaction temperatures of >200 °C. These reactions result in the formation of water, carbon monoxide, and carbon dioxide and influence the hydrogen and carbon content of the biomass.
- The heating value increased from its initial value of about 19.41 MJ/kg to about 23.67 MJ/kg at 270 °C and a 120-min residence time. At lower torrefaction temperatures of 160–180 °C, the increase in the heating value is marginal.

## Figures and Tables

**Table 1:** Methods to measure the chemical properties and higher heating values.

S. No.	Chemical Composition	Procedure
	Proximate	
1	Moisture	ASTM D3173 [29]
2	Ash	ASTM D 3174 [30]
3	Volatiles	ASTM D3175 [31]
4	Fixed carbon	Fixed carbon calculated by the difference method
	**Ultimate Composition**	
1	Moisture	ASTM D3173 [29]
2	Carbon	ASTM D3178 [32]
3	Hydrogen	ASTM D3178 [32]
4	Nitrogen	ASTM D3179 [33]
5	Sulphur	ASTM D3177 [34]
7	Oxygen	Oxygen calculated by the difference method
8	H/C ratio	H/C: number of hydrogen atoms/number of carbon atoms = (%H/1)/(%C/12)
9	O/C ratio	O/C: number of oxygen atoms/number of carbon atoms = (%O/8)/(%C/12)
6	Higher heating value (HHV)	ASTM D5865 [35]

**Table 2:** Experimental design for the deep drying and torrefaction experiments.

Process	Temperatures (°C)	Residence Time (min)	Particle Size (mm)	Heating Rate (°C/min)
Deep drying	160, 180	15, 30, 60, and 120	2	10
Torrefaction	230, 270	15, 30, 60, and 120	2	10

**Table 3:** Proximate, ultimate, and higher heating values of the 2-mm lodgepole pine grind.

S. No.	Chemical Composition	(%)
	Proximate	
1	Moisture	4.2
2	Ash	0.69
3	Volatiles	80.23
4	Fixed carbon	15.1
	Ultimate composition	
1	Carbon	52.23
2	Hydrogen	6.2
3	Nitrogen	0.47
4	Sulphur	0.022
5	Oxygen	41.23
6	H/C	1.42
7	O/C	0.59
8	Higher heating value (HHV)	19.37

**Table 4:** Multiple regression equations for the chemical and energy properties.

S. No.	Chemical Composition and higher heating value	Multiple Regression Equation	Coefficient of Determination (R^2^)	Statistical Significance
	**Proximate composition**			
1	Moisture content (%, w.b.)	$y=5.013-0.0124x_{1}-0.00515x_{2}$	0.93	*p* < 0.001
2	Ash (%)	$y=-0.2718+0.0066x_{1}+0.0029x_{2}$	0.93	*p* < 0.001
3	Volatiles (%)	$y=121.8021-0.2322x_{1}-0.0409x_{2}$	0.79	*p* < 0.001
4	Fixed carbon (%)	$y=-22.9741+0.2236x_{1}+0.0431x_{2}$	0.80	*p* < 0.001
	**Ultimate composition**			
1	Hydrogen (%)	$y=9.4969-0.018x_{1}-0.0054x_{2}$	0.72	*p* < 0.001
2	Carbon (%)	$y=36.3531+0.09716x_{1}+0.0397x_{2}$	0.97	*p* < 0.001
3	Oxygen (%)	$y=54.344-0.0800x_{1}-0.03478x_{2}$	0.96	*p* < 0.001
4	Nitrogen (%)	$y=0.7163-0.00183x_{1}-0.00069x_{2}$	0.89	*p* < 0.001
5	Sulfur (%)	$y=0.03492-0.000071x_{1}-0.000047x_{2}$	0.89	*p* < 0.001
6	H/C ratio	$y=2.158-0.004436x_{1}-0.00179x_{2}$	0.83	*p* < 0.001
7	O/C ratio	$y=1.4845-0.0039x_{1}-0.00096x_{2}$	0.72	*p* < 0.05
8	Higher heating value (MJ/kg)	$y=16.462+0.02321x_{1}+0.0026x_{2}$	0.82	*p* < 0.001

**Table 5:** Analysis of variance for the chemical and energy properties.

S. No.	Chemical Composition and higher heating value	Process Variables	
		Torrefaction Temperature (x_1_)	Torrefaction Residence Time (x_2_)
	**Proximate composition**		
1	Moisture content (%, w.b.)	(−) ***	(−) ***
2	Ash (%)	(+) ***	(+) ***
3	Volatile content (%)	(−) ***	ns
4	Fixed carbon (%)	(+) ***	(+) ***
	**Ultimate composition**		
5	Hydrogen (%)	(−) ***	ns
6	Carbon (%)	(+) ***	(+) ***
7	Nitrogen (%)	(−) ***	(−) **
8	Oxygen (%)	(−) ***	(−) ***
9	Sulfur (%)	(−) ***	(−) ***
10	H/C ratio	(−) ***	(−) *
11	O/C ratio	(−) ***	ns
12	Higher heating value (MJ/kg)	(+) ***	ns

Note: * *p* < 0.05; ** *p* < 0.01; *** *p* < 0.001; ns: non-significant.
